# A new species of *Procambarus* (Decapoda, Cambaridae) from the State of Querétaro, Mexico

**DOI:** 10.3897/zookeys.1048.57493

**Published:** 2021-07-06

**Authors:** Carlos Pedraza-Lara, Pedro Joaquín Gutiérrez-Yurrita, Vladimir Salvador De Jesus-Bonilla

**Affiliations:** 1 Ciencia Forense, Facultad de Medicina, Universidad Nacional Autónoma de México, Circuito de la Investigación Científica s/n, Ciudad Universitaria, Coyoacán, México City, México Universidad Nacional Autónoma de México México City Mexico; 2 Instituto Politécnico Nacional – Centro Interdisciplinario de Investigaciones y Estudios sobre Medio Ambiente y Desarrollo, México City, México Instituto Politécnico Nacional – Centro Interdisciplinario de Investigaciones y Estudios sobre Medio Ambiente y Desarrollo México City Mexico

**Keywords:** Astacidea, Crayfish, integrative taxonomy, species delimitation, systematics

## Abstract

With a Nearctic distribution, the family Cambaridae harbors a high species richness in Mexico, which is also evident along the Pánuco River catchment. A series of surveys carried on in five populations from the Sierra Gorda Biosphere Reserve in the State of Querétaro resulted in localizing a putative new species for science. A molecular phylogenetic study and species delimitation analyses including all the known *Procambarus* species from the Pánuco River catchment were conducted based on three mitochondrial genes (16S rDNA, 12S rDNA, and COI; 2,462 bp in total). Phylogeny recovered all species as monophyletic, including the populations under study. All delimitation results based on barcoding, ABGD, GMYC, bPTP, and gonopod differentiation agree in the recognition of a new taxon, to which the name *Procambarus xihui***sp. nov.** is given, and its diagnosis and description are provided. The new species can be distinguished from the remaining species in the genus, among other characters, by a unique configuration of the terminal elements of the first pleopod of form I male, which includes a central projection lamellate, hood-like, forming a concave blade-like structure mesially directed, as well as a caudal process crest-like, mesiodistally directed, forming a lateral side of the concavity.

## Introduction

The genus *Procambarus* Ortmann, 1905 encompass 45 native species and subspecies occurring in Mexico, inhabiting both Atlantic and Pacific coasts. An important part of the species richness in Mexico inhabits the Pánuco water basin, along the Sierra Madre Oriental and north of the Trans Mexican Volcanic Belt. To date, seven species have been recorded from that region: *Procambarus cuevachicae* (Hobbs, 1941), *P. hidalgoensis* López-Mejía, Álvarez & Mejía-Ortíz, 2005, *P. roberti* Villalobos & Hobbs, 1974, *P. strenthi* Hobbs, 1977, *P. toltecae* Hobbs, 1943, *P. villalobosi* Hobbs, 1969, and *P. xilitlae* Hobbs & Grubbs, 1982. In a survey of the diversity of the genus, previous studies located a series of populations from the aforementioned basin, in the Sierra Gorda Biosphere Reserve, at the northern side of the State of Querétaro, whose specific identity could not be confirmed (Gutiérrez-Yurrita et al. 2002). Analyses by the authors found a degree of morphological distinctiveness compared to other species of the genus from nearby regions while additional studies obtained the 16S rDNA gene from several of them. Later, a study using Random Amplification of Polymorphic DNA and morphological information agreed with the conclusion that several populations from the Sierra Gorda Biosphere Reserve could correspond to an undescribed species (Pedraza-Lara et al. 2004). It is necessary to verify such findings, and to formalize the taxonomic status of such populations (see Materials and methods). Consequently, this article aims to clarify this taxonomic situation using an integrative approach, including molecular markers commonly used for the delimitation of crayfish species in addition to traditional morphology.

## Materials and methods

### Sampling

**Figure 1.**
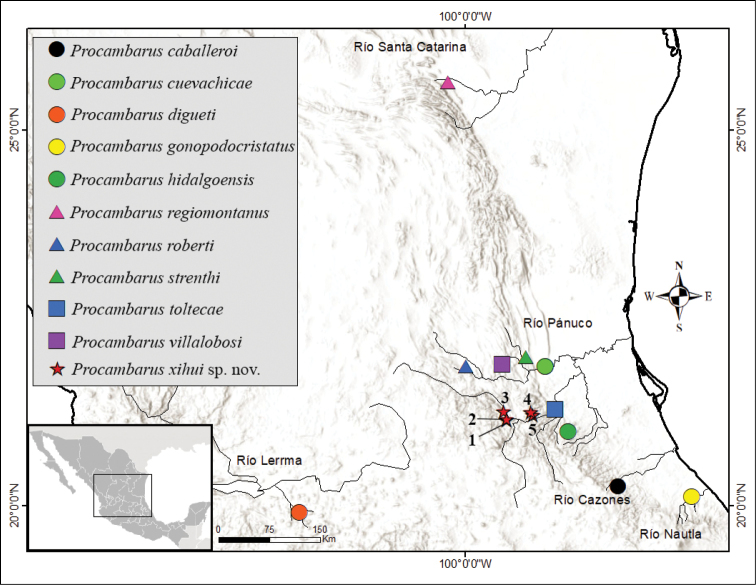
Map of localities. Populations from SGBR are depicted as stars, and numbers correspond to the following sites: 1. Arroyo Los Álamos; 2. Arroyo Camelinas; 3. Saldiveña; 4. Río Verdito; 5. San Juanito.

A series of collections were made in the Sierra Gorda Biosphere Reserve (**SGBR**) for 20 years, beginning in 2002 (Table 1; Fig. 1). This study includes the following populations assigned to the new species from the counties Jalpan de Serra and Landa de Matamoros in the state of Querétaro: Arroyo Álamos, Arroyo Camelinas, Río Verdito, San Juanito and Saldiveña. All were sampled in 2002, 2007, and 2019 by the collectors mentioned in Systematics. For the localities of San Juanito and Saldiveña we failed to obtain any crayfish in 2019. Other populations sampled more than once were Palitla (2002, 2019), Media Luna (2007, 2015, 2019), Santa Anita spring (2012, 2018), and Xicotepec (two occasions in 2019). Specimens were collected by hand and identified using the available keys (Hobbs 1972a), original descriptions, and reviews (Hobbs 1941, 1943, 1977; Villalobos 1944, 1955, 1958; Villalobos-Figueroa 1954; Villalobos Figueroa and Hobbs 1974; López-Mejía et al. 2005). Details of the collection sites are provided in Table 1. Type material was deposited at the following Institutions: National Collection of Crustaceans, Institute of Biology, Universidad Nacional Autónoma de México (**CNCR**); National Museum of Natural History, Smithsonian Institution, USA (**NMNH**) and Arthropod Collection of Forensic Reference at Forensic Program, UNAM, Mexico (**CARF**). The holotype (CNCR 35721), allotype (CNCR 35723), and morphotype (CNCR 35722) were deposited at CNCR. Paratype series (one male form I, and one female, under catalog number USNM 1638484 and USNM 1638485, respectively) were deposited at NMNH. Additional paratypes were deposited at CARF under catalog numbers CARF – CPLC45 – CARF – CPLC47. Measurements of the types are provided in Table 2.

**Table 1.** Species, locality data, and GenBank accession numbers of specimens used in the phylogenetic and species delimitation analyses.

Species	Locality	Specimen	Collection year	GenBank accession numbers		
				16S	12S	cox1
*Procambarus xihui* sp. nov.	Arroyo de Los Álamos, Yerbabuena, Jalpan de Serra, Querétaro *	CPLC1†	2019	MW280269	MW280231	MW266807
	Arroyo de Los Álamos, Yerbabuena, Jalpan de Serra, Querétaro *	CPLC23‡	2019	MW280277	MW280238	MW266814
	Arroyo Camelinas, Yerbabuena, Querétaro	CPLC27	2002	MW280280	MW280242	MW266816
	San Juanito, Landa de Matamoros, Querétaro	CPLC24	2002	–	MW280239	–
	Río Verdito, Landa de Matamoros, Querétaro	CPLC25	2019	MW280278	MW280240	–
	Saldiveña, Jalpan de Serra, Querétaro	CPLC26	2007	MW280279	MW280241	MW266815
*P. toltecae*	Stream 1 Km Soutwest of Palitla, San Luis Potosí*	CPLC3	2019	MW280270	MW280246	MW266808
	Stream 1 Km Soutwest of Palitla, San Luis Potosí*	CPLC28	2019	MW280281	MW280243	MW266817
	Huichihuayán, San Luis Potosí**	PopHui	2012	JX127823	JX127687	JX127966
*P. hidalgoensis*	Stream on driveway from Tlanchinol-Olotla, Hidalgo	CPLC5	2019	MW280272	MW280233	MW266810
	Stream on driveway from Tlanchinol-Olotla, Hidalgo	CPLC29	2019	MW280282	MW280244	–
*P. villalobosi*	Cave East of Rayón, San Luis Potosí*	CPLC11	2019	MW280274	MW280235	MW266812
*P. villalobosi*	Cave East of Rayón, San Luis Potosí*	CPLC33	2019	MW280285	–	MW266820
*P. gonopodocristatus*	María de la Torre, Veracruz*	CPLC30	2019	MW280283	–	MW266818
	María de la Torre, Veracruz*	CPL2474	2019	MW280268	MW280230	–
*P. roberti*	Creek coming from La Media Luna, 0.5 Km East, San Luis Potosí*	CPLC13	2019	MW280276	MW280237	–
*P. roberti*	Creek coming from La Media Luna, 0.5 Km East, San Luis Potosí*	CPLC32	2007	MW280284	MW280245	MW266819
*P. roberti*	–***	roberti1	–	KX238070	–	–
*P. strenthi*	Santa Anita spring, San Luis Potosí*	CPLC10	2018	MW280273	MW280234	MW266811
	–***	strenthi1	2017	KX238078	–	–
*P. caballeroi*	Stream southern of Xicotepec de Juárez, Puebla*	2419	2019	MW280265	MW280226	MW266803
	Stream southern of Xicotepec de Juárez, Puebla*	2420	2019	MW280266	MW280227	MW266804
	–***	Pcb302	–	KX238005	–	–
*P. cuevachicae*	La Cueva Chica, Ciudad Valles, San Luis Potosí*	2424	2020	–	MW280228	MW266805
	La Cueva Chica, Ciudad Valles, San Luis Potosí*	2425	2020	MW280267	MW280229	MW266806
*P. acutus*	Canal en Ciudad Mante, Tamaulipas	3952	2007	MW280264	–	MW266802
	Canal en Ciudad Mante, Tamaulipas**	PopMan	2007	JX127827	–	JX127970
*P. digueti*	Camécuaro River, Michoacán	CPLC12	2012	MW280275	MW280236	MW266813
*P. regiomontanus*	Guadalupe, Nuevo León	CPLC4	2018	MW280271	MW280232	MW266809
	***	DJ43	2018	KX238068	KX238138	KX238224

* Type locality; ** from Pedraza-Lara et al. 2012; *** from Stern et al. 2017; † holotype; ‡ allotype.

**Table 2.** Measurements of types. Morphometric measurements (mm) of holotype, allotype, and morphotype of *P. xihui* sp. nov.

Measurements	Holotype	Allotype	Morphotype
Total Length (TL)	59.80	61.41	65.68
Cephalothorax			
Length (CL)	28.89	28.99	32.15
Height (CH)	13.91	14.11	15.63
Width (CW)	13.71	13.99	15.26
Cephalon length (CEL)	18.95	19.75	21.29
Abdomen width (AW)	12.08	11.82	13.21
Rostrum			
Length (RL)	7.26	7.18	8.60
Width (RW)	4.85	5.59	5.65
Acumen length (AL)	1.39	1.03	1.77
Antennal scale length (ASL)	6.11	6.41	6.86
Cheliped			
Chela length (CHL)	25.75	19.22	27.82
Chela width (CHW)	8.02	5.98	7.84
Dactyl length (DL)	14.50	11.30	16.01
Palm length (PL)	9.56	7.12	8.81
Merus length (ML)	13.13	11.38	13.32
Areola			
Areola width (ARW)	9.12	8.79	10.56
Areola length (ARL)	2.00	2.05	2.51

Aiming to account for an accurate representation of Mexican species of *Procambarus*, except for the troglobitic *Procambarus xilitlae*, all the species previously assigned to the subgenus Ortmannicus (Hobbs 1972b) were included: *P. acutus*, *P. caballeroi*, *P. cuevachicae*, *P. gonopodocristatus*, *P. hidalgoensis*, *P. toltecae*, and *P. villalobosi*. In addition, *P. digueti* and *P. regiomontanus* were included as outgroups for phylogenetic analysis and reference in species delimitation (Table 1). Voucher numbers were assigned to all specimens and included in CARF (UNAM). Laboratory work was carried on in the Forensic Entomology Lab, Forensics Program, at UNAM.

Specimens were identified using the appropriate taxonomic keys (Hobbs 1972a; Bezerra et al. 2020), as well as the respective taxonomic descriptions. Measurements of type specimens were made on standard morphological characters used in crayfish taxonomy (Pedraza-Lara and Doadrio 2015). For paired characters, measurements were taken from the left side of the specimen with a digital caliper (Mitutoyo’s Absolute Series 500, resolution: 0.01 mm) and a stereoscopic microscope (Leica M60 APO). A validation procedure was done by measuring the same randomly chosen individuals three times. A Pearson correlation between replicates was done in which values above 0.8 were considered as indicatives of low measurement error. This was verified and all measurements, which showed values above 0.8. Drawings from type series for description were prepared by direct observations using a caliper and a stereoscopic microscope (Leica M60 APOP) by Aslam Narvaez. Additionally, SEM pictures were taken from gonopods, epistome, and antennal scale of holotype, as well as annulus ventralis from allotype, at the Electronic Microscopy Laboratory, IBUNAM.

### Phylogenetic and species delimitation analysis

Specimens were preserved in ethanol and a piece of abdominal muscle was taken for DNA purification, which was carried on using a phenol-chloroform protocol (Sambrook et al. 1989). Three mitochondrial genes were sequenced: 16S rDNA (16S), 12S rDNA (12S), and Cytochrome Oxidase subunit I (COI). These genes have accurate phylogenetic signal in crustaceans and are considered optimal choices to characterize the genetic variation and species delimitation in crustacean groups (Toon et al. 2009; Matzen da Silva et al. 2011; Pedraza-Lara et al. 2012). PCR amplifications using gene-specific primers were done using primers and following conditions previously standardized on cambarid species delimitation (Pedraza-Lara and Doadrio 2015) (see Table 3 for details on amplifications and genes analyzed). Amplifications were carried out in 10 mL reactions containing: 1X PCR buffer, 0.5 mM of each primer, 0.2 mM of each dNTP, 1.5 mM MgCl2, 1 U Platinum Taq polymerase (Thermo), and 10–50 ng of template DNA.

**Table 3.** Variability parameters of analyzed gene fragments and the most accurate substitution models.

Gene	Primers*	bp	V	PI	Model**
16S	1471	559	169	82	GTR+G
	16S-1472				
12S	12sf	397	71	51	GTR+I+G
	12sr				
COI	ORCO1F	1506	221	138	GTR+I+G
	ORCO1R				

bp = length in base pairs; V = variable sites; PI = parsimony informative sites; * amplification conditions followed Pedraza-Lara et al. 2012; ** most appropriate substitution model selected using Partition Finder 2.

To investigate the species limits between the putatively undescribed taxon and other *Procambarus* species with molecular information we used the following approaches: genetic divergence of the barcoding COI gene (Hebert et al. 2003), the Automatic Barcode Gap Discovery (ABGD) (Puillandre et al. 2012), Bayesian Tree Poisson Process (bPTP) (Zhang et al. 2013), and the General Mixed Yule Coalescent model (GMYC) (Pons et al. 2006). Barcoding and ABGD analyses were carried using the COI gene; input for bPTP and GMYC was a concatenated matrix with the three mitochondrial markers.

The uncorrected *P*-distances and standard error of the COI marker between putative species were calculated in Mega 10.1.8, estimating standard error based on bootstrapping (Kumar et al. 2018). To determine the barcode gap, the ABGD analysis was run online (https://bioinfo.mnhn.fr/abi/public/abgd/abgdweb.html) setting the simple distance (relative gap width) to 0.5, and default values for the remaining parameters.

For the GMYC approach, an ultrametric tree was reconstructed in Beast 2.6.2 (Bouckaert et al. 2014) using the GTR + Γ + I model, a relaxed clock lognormal, and Yule model prior. Bayesian Markov chain Monte Carlo was run for 25 million generations, sampling trees every 1,000 generations. The log file was inspected in Tracer 1.7.1 (Rambaut et al. 2018) to confirm convergence and Effective Sample Size (ESS) ≥ 200. A single maximum credibility tree was summarized with TreeAnnotator v2.6.2 after removing 15% of the trees as burn-in. The resulting tree was used as input to delimit species with the single threshold GMYC approach in the package ‘splits’ implemented in R (http://r-forge.r-project.org/projects/). The bPTP analysis was performed online (https://species.h-its.org/ptp/) using a ML phylogenetic tree as input (see below), the analysis was run 100,000 MCMC generations with burn-in of 15%.

A phylogenetic hypothesis regarding the included specimens of *Procambarus* species was reconstructed with Maximum Likelihood (ML) and Bayesian Inference (BI) methods. These analyses were carried out to evaluate the congruence of the delimitation analyses previously mentioned with the formation of monophyletic clades at the terminals and evaluate its clade support. Conformation to monophylly is also another way to assist during taxon recognition (Rosen 1979; Donoghue 1985). The ML reconstruction was conducted in RAxML 8.2.12 (Stamatakis 2014) with a rapid bootstrap algorithm (-f a) with 1000 bootstrap replicates. For the BI method, the appropriate substitution model for each marker was inferred with Partition Finder 2 (Lanfear et al. 2017). The BI reconstruction was conducted in MrBayes 3.2.7a (Ronquist and Huelsenbeck 2003). We ran two runs with four MCMC chains with 50,000,000 generations, sampling every 1000 generations and setting a burn-in of 10%. Convergence of chains and ESS (> 200) were confirmed in Tracer 1.7.1 (Rambaut et al. 2018).

It has been described that habitats in the SGBR face important threats like increasing drying (Mendoza-Villa et al. 2018), the cutting of forests, introduction of exotic species, destruction of habitat for agriculture and grazing, pollution of water and the alteration of river channels for human activities (Gutiérrez-Yurrita 2014). Considering this, an assessment of the extinction risk was done using the International Union for Conservation of Nature (IUCN) Red List of Threatened Species Categories and Criteria (IUCN Standards and Petitions Committee 2019). IUCN criteria were applied to the crayfish populations inhabiting the SGBR.

The data underpinning the analysis reported in this paper are deposited at GBIF, the Global Biodiversity Information Facility, and are available at https://doi.org/10.15468/3hu4bh.

## Results

In all cases, morphological features were congruent and stable when several form I male specimens were available for one species. No issues were evident when separating and identifying species according to the literature. As usual in *Procambarus*, the morphology of the first pair of pleopods of male form I was useful to identify and distinguish the new species, as the structure of terminal elements was always congruent with what was originally described and allowed robust species identification. Accordingly, a series of unique traits were observed for the populations from the SGBR.

### Species delimitation and phylogenetic analyses

The following gene fragments were obtained: 16S (559 bps), 12S (397 bps), and COI (1506 bps), resulting in 2462 characters and giving a series of substitution models (Table 3). The resulting sequences were deposited in GenBank (Table 1), no signs of numts were found. The most variable fragment was 16S, followed by 12S and COI (variable sites: COI = 221/1506, 16S = 169/559 12S = 71/397); besides this, COI showed the highest proportion of parsimony-informative (PI) sites: COI = 138, 16S = 82, 12S = 51 (Table 3).

**Figure 2.**
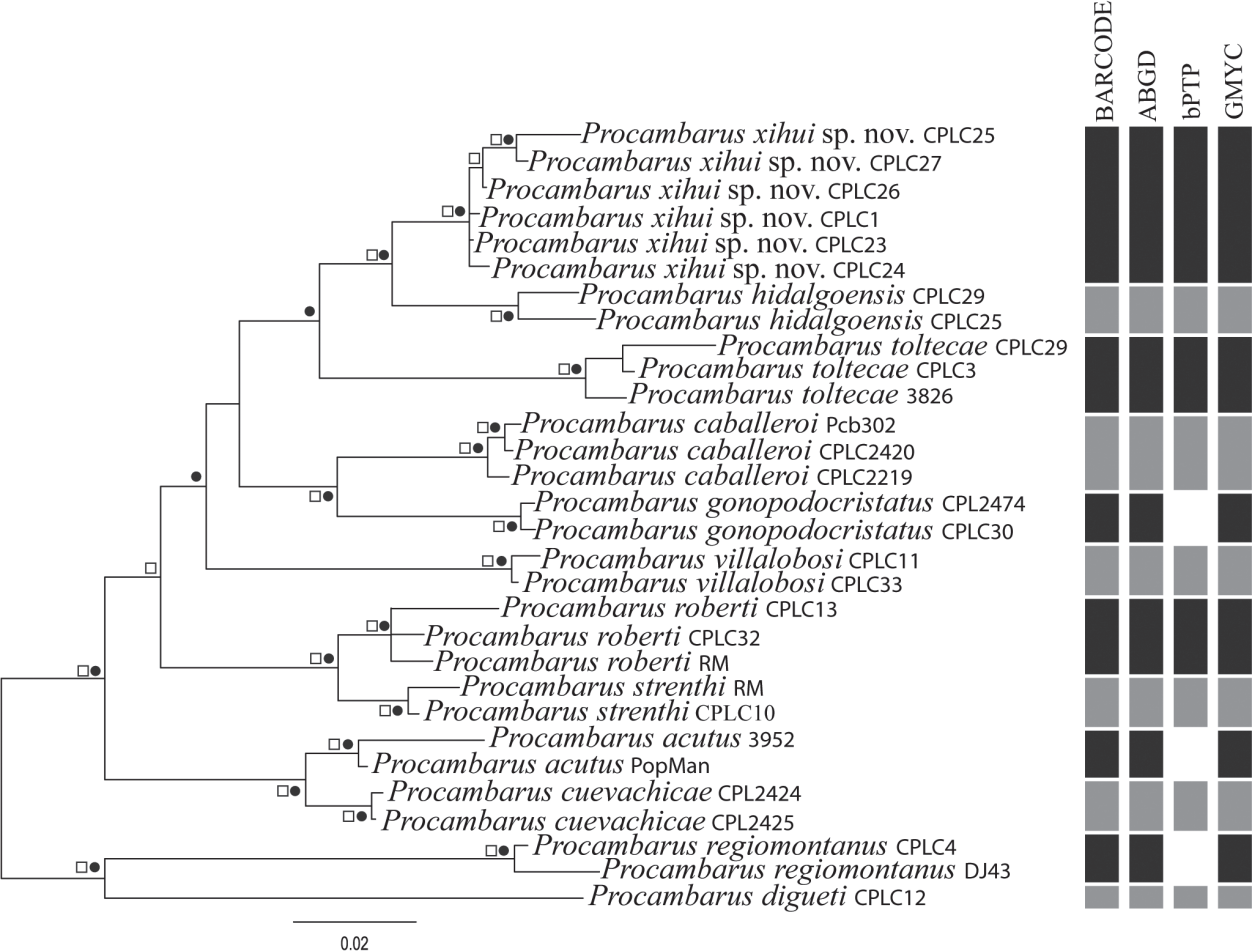
Phylogenetic tree of species analyzed. Codes are referred to in Table 1. BI and ML topologies were completely congruent. Support values of 95 or more (BI) and of 70 or more (ML) are depicted with figures above nodes; circles = posterior probabilities, squares = bootstrap support. At right, recovery of species delimited according to each of the four delimitation criteria used.

All delimitation analyses recovered a congruence between morphological identifications and molecular information for all species (Fig. 2; Suppl. material 1). The populations from SGBR are delimited as one distinct species according to all delimitation criteria, including the morphological observations carried on over specimens sampled. The COI genetic *P*-distance between all localities of the SGBR and the other species of *Procambarus* ranges from a minimum of D_p_ = 3% for that observed with *Procambarus hidalgoensis*, and a maximum of D_p_ = 10.6% of with *Procambarus regiomontanus* (Suppl. material 2: Table S1). Such values are above D = 1% and above what is common for between-species distances in crustaceans and crayfish (Fetzner and Crandall 2002; da Silva et al. 2011).

Congruently, the ABGD analysis recovered the undescribed taxon as a separate species from other *Procambarus* species. The bPTP and GMYC species delimitation analyses separate sequences of such populations as a distinct taxon; GMYC confirms the latter observation as well as the specific status of the remaining *Procambarus* species (Fig. 2). With the most supported partition scheme of bPTP analysis, all specimens assigned to *Procambarus gonopodocristatus*, *P. acutus*, and *P. regiomontanus* were not supported as forming one species each; however, the estimated number of species considered by bPTP is between 10 and 26, which includes the scheme of species delimited by the other methods. Bayesian inference and maximum likelihood topologies were congruent (Fig. 2). Topology recovered all species as monophyletic in highly supported clades. One clade included *Procambarus acutus* and *Procambarus cuevachicae*; next, a clade is recovered containing the remaining species from the Pánuco basin except for *Procambarus caballeroi* and *Procambarus gonopodocristatus*, inhabitants of distinct basins south of the Trans Mexican Volcanic Belt (TMVB). Inside this clade, the populations from SGBR were included in a clade in a sister relationship to *P. hidalgoensis*. These both form a clade close to populations of *P. toltecae*. In the light of these results, a new species is described which includes populations analyzed from the SGBR.

Regarding the conservation assessment, in total, five populations for the species were recorded: populations from Álamos and Camelinas fall into one single 5–10 km^2^ quadrant, and Saldiveña, Río Verdito, and San Juanito each falls into their own 5 km^2^ quadrant. This resulted in a maximum area of occupancy of 25–35 km^2^. However, this would be extremely inaccurate, as the available area of habitat (small streams, probably fragmented by large-magnitude creeks) is much more reduced inside each quadrant. Consequently, we consider that a gross estimation of area of occupancy for the species would fall in less than 5 km^2^. Considering the factors aforementioned, we found a conservation status for *Procambarus xihui* of Critically Endangered (CR) based on the following criteria: B.2.*a* (habitat severely fragmented), B.2.*b.ii* (continuing decline in area of occupancy), and B.2.*b.iv* (continuing decline in number of locations).

### Systematics

**Cambaridae Hobbs, 1942**

#### Genus *Procambarus* Ortmann, 1905

##### Procambarus xihui sp. nov.

Taxon classificationAnimaliaDecapodaCambaridae

0E5B74BC-5332-5728-BA98-561A1D793372

http://zoobank.org/DCFCDB8F-896F-4071-8CB6-12D6241FE9DB

**Figure 3.**
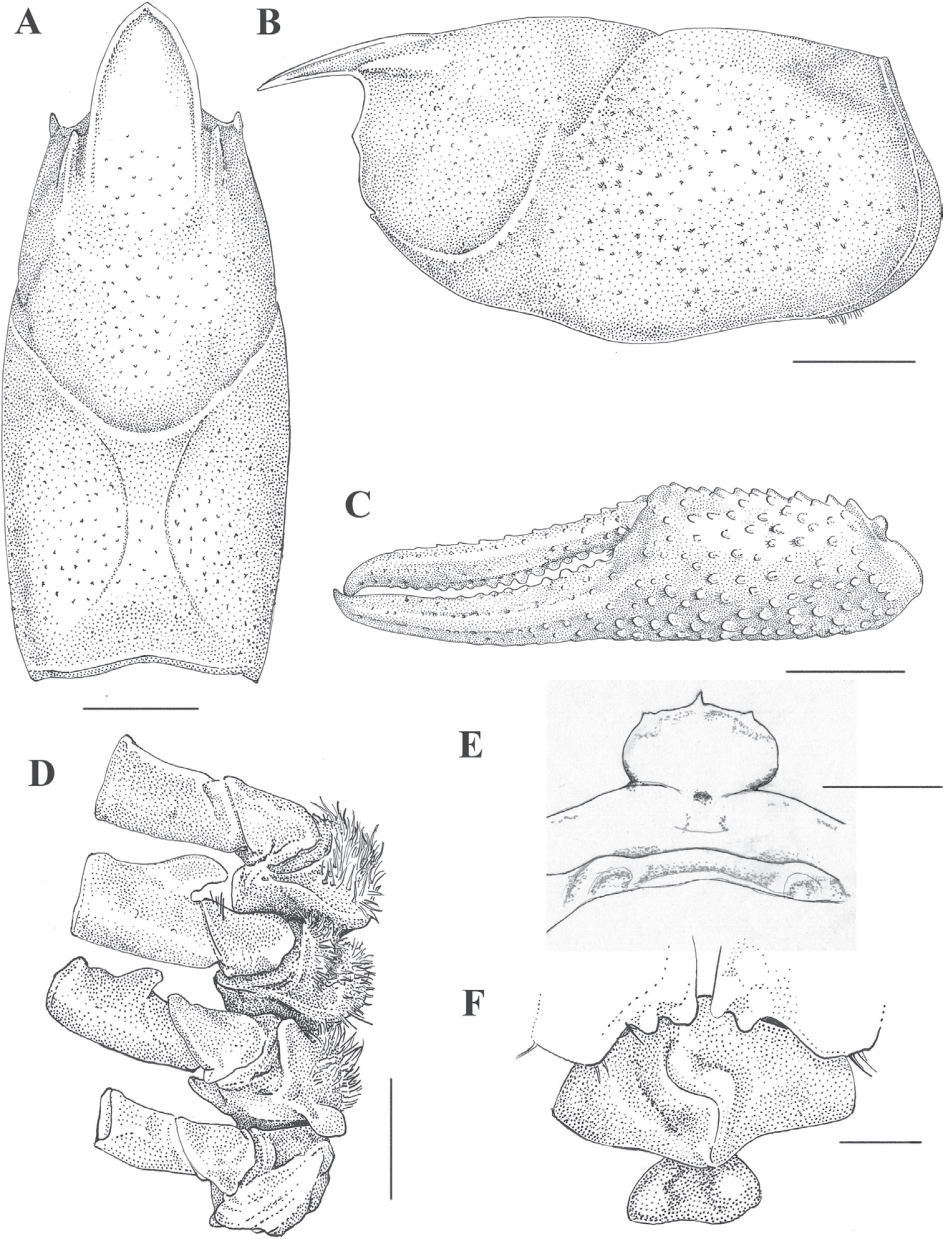
*Procambarus xihui*. All illustrations from holotype except for F which is from allotype **A** dorsal view of cephalothorax **B** lateral view of cephalothorax **C** lateral view of cheliped **D** basal podomeres of second to fifth pereiopods **E** Epistome **F** caudal view of annulus ventralis. Scale bars: 5 mm (**A–C**); 2 mm (**D–F**).

**Figure 4.**
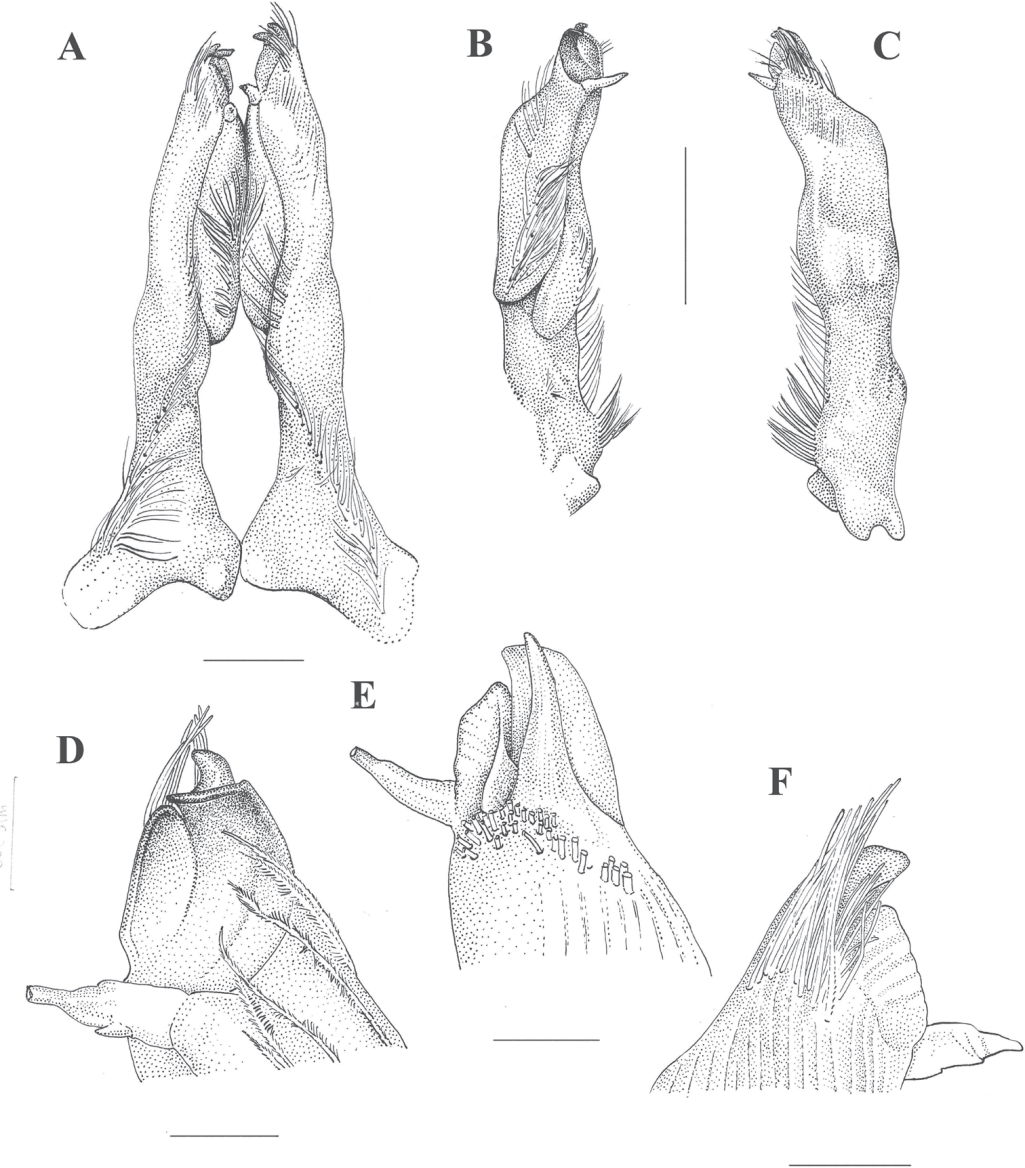
*Procambarus xihui*, holotype **A** caudal view of the first pair of pleopods **B** mesial view of left gonopod **C** lateral view of left gonopod **D** detail of apex, mesial view **E** detail of apex, latero-cephalic view **F** detail of apex, lateral view. Scale bars: 2 mm (**A**); 0.5 mm (**B, C**); 0.1 mm (**D–F**).

Figures 3 , 4 , Table 2

###### Material examined.

***Holotype:*** male from I (CNCR 35721), 21°8.548'N, 99°17.106'W, ca 1210 m; stream Los Álamos, Yerbabuena, Jalpan de Serra, Querétaro State, Mexico. A small headwater first-magnitude stream, which keep water in shallow ponds along the year. leg. Heriberto Pedraza Rodríguez, Patricia Ornelas-García, Carlos Pedraza-Lara, Ma. Guadalupe Lara Zúñiga, Guadalupe Gracia, Regina Pedraza Lara, May 22, 2019. ***Allotype***: female (CNCR 35723), same data as holotype. ***Morphotype***: male (CNCR 35722), same data as holotype.

###### Diagnosis.

Body pigmented, eyes well developed. Rostrum lanceolate, concave, without lateral spines; antennal scale width 0.50–0.54 × in its length; areola of moderate width (0.22–0.23 × wide in length) with 2–4 large punctations in narrowest part; cervical spine absent, single, shallow branchiostegal spine; chela shorter than cephalothorax length, long and thin, length 0.87–0.89 × the length of cephalothorax and 0.28–0.31 × wide than long, narrow-ovate. Dactyl forming a concave profile in mesial margin, palm of chela with scattered tubercles, mesial surface with row of seven or eight tubercles, palm length 0.55–0.66 × in dactyl length; no lateral spines on carapace; postrostral ridges very strong and wide, forming a strong tubercle, provided with longitudinal groove along its laterodorsal margin, its apical extreme slightly overreaching carapace surface, not forming evident apical spine. Male with hooks on ischiopodites of the third and fourth pairs of pereiopods, those on third ischiopodite extending beyond basioischial articulation.

First pair of pleopods slightly asymmetrical, reaching coxopodite of third pereiopod, with shoulder on cephalic margin beginning at distal fifth; a row of setae from base to second third of pleopod, a second row of setae along mesial surface starting at mid-length and third row of setae along mesial surface starting on last quart and extending laterally to base of terminal processes, where it forms a tuft of plumose setae; mesial process spiniform, directed caudally and slightly mesially, cephalic process spiniform, acute, hood-like, directed caudomedial, upon central projection and hidden beneath apical tuft; central projection corneous, lamellate, hood-like, tip decidedly projecting mesially, forming a concave blade-like structure, distally folded in mesial direction and reaching beyond the remaining terminal elements; caudal process corneous, crest-like, running on caudomesial surface of pleopod tip, along longitudinal pleopod axis, mesiodistally directed, forming a lateral side of the concavity formed distally by the central projection, reaching bellow point of mesial process position in lateral view.

Preanular plate with strong tubercles in caudal margin, and with setae along its margin, both well projecting over annulus cephalic area. Annulus ventralis rather fusiform, with depression along median surface and sinus in shallow Z-shape. Endopodite and exopodite of uropods with strong distolateral spines and median ridge ending in small spine, not reaching endopodite margin.

###### Description of holotypic male, Form I.

(Figs 3, 4, Table 2). Body pigmented, eyes well developed. Body subovate, abdomen narrower than thorax. At cervical groove carapace slightly higher than wide (0.99 × height). Areola moderate in width (0.22 × length) with three or four punctations in narrowest part; length of areola ca. 0.32 × that of entire carapace length. Rostrum lanceolate, dorsally excavated, reaching distal third of second basal segment of antennule, its width 0.69 × in length; margins raised slightly thickened, acumen not sharped, dorsal surface of rostrum punctuated at its base, row of setiferous punctations along base of marginal ridges, subrostral ridges poorly developed, and not evident from dorsal view.

Postrostral ridges conspicuous and wide, forming a strong tubercle, provided with longitudinal groove along its laterodorsal side, its apical edge slightly overreaching carapace surface, not forming evident apical spine. Suborbital angle obtuse, one branchiostegal spine present. Surface of the carapace deeply punctuate.

Epistome broadly triangular, subsymmetrical, with cephalomedian projection well defined. Antennule with ventral spine on basal segment well developed. Antennal scale width 0.5 × its length, maximum width at ca. 0. 5 × length, with a ridge along lateral margin ending in a strong spine.

Chela long and thin, 0.89 × the length of carapace and 0.31 × wide as long, narrow-ovate, dactyl forming a concave profile in mesial margin. Chela scattered with numerous setose tubercles and crowded with numerous denticles. Mesial margin of palm with row of seven tubercles, opposable sides of both fingers with strong tubercles, seven stronger on proximal half of dactyl. Fingers gaping along their length. Lateral margin of dactyl with weak ridge of acute tubercles proximally and punctations distally. Tip of fingers forming strong pencils. Opposable margin of fixed finger with four tubercles on basal one-quarter and five punctations along second and third distal quarters.

Width of carpus of first pereiopod ca. 0.63 × in its length. Merus length 0.45 × in cephalothorax length, with scattered punctations in lateral surface, two rows of spike-like tubercles on mesial surface, stronger at distal half, apical spine present. Hooks on ischiopodites of third and fourth pereiopods, former well exceeding basioischial articulation, latter reaching it. Bases of coxopodites of fourth and fifth pereiopods with caudomesial boss projection, the former extending on wide prominence on caudoventrally surface, caudomedial oriented, setose around margin, the latter blade-like, mesially oriented, bare.

###### First pleopods as described in diagnosis.

Abdomen slightly narrower than carapace, width 0.88 × in cephalothorax width. Protopodite of uropods with distolateral spines, endopodite and exopodite with strong distolateral spines and median ridge ending in small spine, not reaching endopodite margin. Dorsal side of telson with one median spine on each caudolateral corner.

###### Description of allotypic female.

(Fig. 3, Table 2). Differing from holotype in following respects: areola of moderate width (0.23 × length) with two or three punctations in narrowest part, areola length 0.3 × carapace length. Rostrum wide (0.78 × rostrum length).

Shorter and smaller chela, 0.66 × length of carapace and width 0.31 × length, mesial profile of dactyl straight. Four strong tubercles on proximal half of opposable side of dactyl. Two conspicuous tubercles on opposable side of fixed finger, one on distal third. Width of carpus of first pereiopod ca. 0.63 × its length. Shorter merus, 0.39 × cephalothorax length. Left dactyl abnormally small, shorter than fixed finger. No hooks on ischiopodites of pereiopods. Caudomesial boss only evident on fifth coxopodite, mesially projected.

Annulus ventralis as described in diagnosis (Fig. 3). First pleopods uniramous, reaching cephalic region of annulus ventralis when abdomen is flexed.

###### Description of morphotypic male, form II.

(Table 2). Differing from holotype in the following respects: areola of moderate width (0.24 × length) with punctations (two or three in narrowest part).

Left chela 0.87 × the length of cephalothorax and width 0.28 × in its length, mesial surface of chela with a row of ten tubercles, palm 0.55 × in dactyl length. Right chela abnormally smaller. Opposable side of dactyl with five stronger tubercles on proximal side, lateral margin of dactyl with ridge of punctations. Opposable margin of fixed finger with five tubercles on basal quarter, two of them stronger, and punctate along distal half.

Carpus of first pereiopod ca. 1.35 × longer than wide. Shorter merus (0.41 × cephalothorax length). Shallow hooks on ischiopodites of third and fourth pereiopods, the former longer, none exceeding basioischial articulation.

Terminal elements of first pleopods not stylized, certain incipient development in mesial process and central projection, the latter together with caudal and cephalic processes mesially oriented.

**Figure 5.**
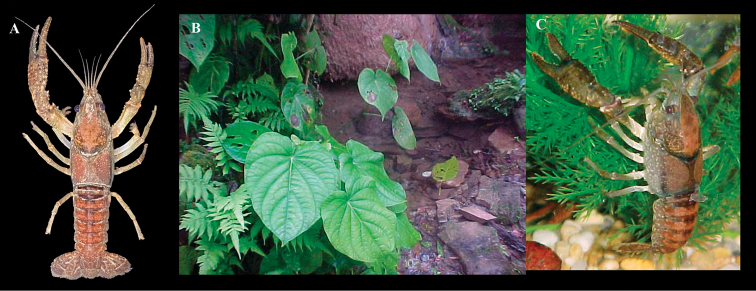
*Procambarus xihui***A** photograph of form I male alive showing coloration **B** general habitat in type locality **C** photograph of live specimen in aquarium. Photographs by CPL.

The new species depicts certain variability in coloration among populations, but most individuals show a general brownish body background with lighter scattered spots along thorax and abdomen (Fig. 5). For most individuals, the chela is brown to reddish, with scattered darker or yellowish punctations. Color become lighter to the base of pereiopods. In some individuals, a diffuse darker band is visible on the sides of thorax, which become darker posteriorly, but it is not apparent in others.

###### Etymology.

The specific epithet -*xihui* comes from the term used by natives from the region, (also known as the Pame people), to refer to themselves. The term also means ‘indigenous’ in the Pame language.

###### Phylogenetic relationships and remarks.

Except for *Procambarus digueti* and *P. regiomontanus*, which are clearly distinctive among the crayfish fauna of Mexico and used here as outgroups, the new species shares some traits with the remaining species included, most of them inhabiting the Pánuco River basin. Among those are the possession of hooks on the ischiopodites of third and fourth pereiopods and the first pair of pleopods reaching the coxa of third pereiopods. However, the new species can be readily distinguished from two other species included inhabiting the Pánuco basin, *P. strenthi* and *P. roberti*, based in the following characters (among others): in *P. roberti*, the first pleopods are asymmetrical and lack a cephalic shoulder, and it possess a subtriangular, laterally grooved caudal process abutting the caudal base of central projection, which is notably more reduced than the shown by *P. xihui*. In *P. strenthi*, the first pleopods of the male form I are also strongly asymmetrical, bearing a strong angular shoulder in the cephalic surface, a cephalic process broad and lamellate, a dentiform central projection and a smaller subtriangular caudal process.

More specifically, the new species is morphologically related to a group of species placed in the subgenus Ortmannicus by Hobbs (1972), although subgeneric groupings in *Procambarus* have not been recognized recently (Crandall and De Grave 2017). Still, such grouping allows us to identify some morphological similarities among *P. xihui* and the species morphologically most like it. Such species are *P. acutus*, *P. caballeroi*, *P. cuevachicae*, *P. gonopodocristatus*, *P. hidalgoensis*, *P. toltecae*, and *P. villalobosi*. Several traits are shared among the new species and the remaining Mexican species assigned to *Ortmannicus* sensu Hobbs (1972) such as the lack of caudal knob. In general, the new species can readily be distinguished from the remaining species by the configuration of terminal elements of the first pair of pleopods. In addition, it can be distinguished from *P. acutus* and *P. cuevachicae* as these show a distally directed mesial process, a cephalic process somewhat rounded distally, an acute caudal process, a somewhat twisted central projection, and an almost obliterated areola. *P. acutus* and *P. cuevachicae* also lack a cephalic shoulder in the first pleopod. The new species can be readily separated from *P. villalobosi*, among several other traits, by the conspicuous arrangement of all apical elements of the first pleopod in *P. villalobosi*, which has a singularly long mesial process far exceeding the other elements caudally.

Among other differences, the new species can be separated from *P. caballeroi* as the latter possess a wider rostrum, a laminated, laterally flattened cephalic process, a crest-like caudal process whose apex ends in a spine-like structure that is caudodistally directed. Among the main differences with *P. gonopodocristatus* are that the latter possesses a caudal process in the form of a long blade arced along the caudolateral surface, when in *P. xihui* this process is longer and situated along the caudomesial surface of the pleopod. *Procambarus caballeroi* and *P. gonopodocristatus* inhabit other river basins, south of the TMVB. The two species that most resemble *P. xihui* are *P. toltecae* and *P. hidalgoensis*.

The new species can easily be differentiated from *P. toltecae* because the latter shows a different arrangement of the terminal elements of the first pleopod: most conspicuous are the caudal orientations of the cephalic and caudal processes as well as the central projection, the latter two forming a triangular projection which extends in caudally and forms a right angle to the longitudinal axis of the appendix. In *P. toltecae*, the central projection is the longest among the related species, while in *P. xihui*, the three most apical elements are directed mesially and the caudal process is blade-like and runs along the mesial side of the pleopod. We find that the new species is most similar to *P. hidalgoensis*, from which, however, clear differences can be noticed. In the latter, the mesial process is latero-distally oriented, while in *P. xihui* its orientation is caudal and slightly mesial; both show a central projection that is corneous and flattened, but its division in two elements in *P. hidalgoensis* is clear, one larger and distally projected and the other shorter, straight, and mesially projected, while in *P. xihui* the two elements are fused and no clear delimitation exists between them unless observed on electron microscopy; they form one concave blade-like structure, distally folded in a mesial direction. The caudal process is laminated in both species, but in *P. hidalgoensis* it is located mesiocaudally to central projection, while in *P. xihui* it is more laterally located, becoming the lateral side of the concavity formed by the central projection, also mesially directed. In vivo, a distinctive red coloration was recorded in the male form I of *P. hidalgoensis* with a contrasting blackish stripe running laterally of cephalothorax. In *P. xihui*, a dark stripe can be present, but it does not contrast as the body color is brownish (Fig. 5).

The phylogenetic analysis partially agrees with deductions from morphological similarities. The new species is grouped in a clade with *P. hidalgoensis*: these two species inhabit small, first-order springs of the Pánuco basin, although *P. xihui* inhabits higher altitude parts of three different sub-basins (between 1,000 m and ca. 1,200 m): the Jalpan River (later a tributary of the Santa María sub-basin), the Tancuilín sub-basin, and Extoraz sub-basin (both tributaries of the Moctezuma River). On the other side, *P. hidalgoensis* inhabits similar habitats (at an altitude of 1,485 m) but from the Río Hule sub-basin, a southern component of the Moctezuma sub-basin. This clade is grouped with *P. toltecae*, which inhabits much lower altitudes (here collected from 273 m). Similarly, the Pánuco system is inhabited by the remaining species here included except for *P. digueti* and *P. regiomontanus*, but most of them are from distinct sub-basins or altitudes. Results shown here support that this region is a depositary of distinct clades of crayfish diversity in Mexico, which possibly reflects a complex biogeographic history for the genus in northeast Mexico, from which *P. xihui* is one additional component. Additional phylogenetic and biogeographic inferences are surely complex and beyond the scope of the present manuscript and will be treated in further work.

###### Habitat and conservation notes.

The new species inhabits an entirely included area in the SGBR. With certain variation among populations, habitats are headwater stream ecosystems, less than 1.5 meters wide, showing surface water intermittently along their course for most of the year, especially in small ponds that are 0.5–3 m wide with reduced water flow (Fig. 5). These are very sensitive habitats, reduced in area and characterized by a high quality of riparian vegetation and pristine water conditions (Meyer et al. 2007). During the rainy season they can occasionally join the next water course, where crayfish populations have not been found; consequently it is possible that a high degree of habitat fragmentation can exist between locations. They are characterized by oligotrophic water conditions (elevated oxygen concentration, low temperatures and low nutrients) and substrates composed of bedrock, rocks, pebbles, cobbles, leaf litter, tree branches (Pedraza-Lara et al. 2004), and other elements that provide shadow, refuge, and high habitat heterogeneity. The riparian vegetation, rocks, and gravels are of special importance for crayfish survival since they are nocturnal and usually spend most of the day hidden in these substrates.

The characteristic physical and chemical parameters of their habitats are temperatures between 20 and 28 °C, dissolved oxygen content between 8 and 12 mg l^-1^, pH 7–8 , and water hardness 90–350 mg CaCO3 l^-1^. The terrestrial vegetation of the riverside where the crayfish populations were found is composed by riparian vegetation of *Platanus mexicana*, *Taxodium mucronatum*, and *Salix* species.

Headwater streams might be more vulnerable to disturbances in the surrounding catchment than other aquatic habitats, which relate to a higher risk of biodiversity loss (Lowe and Likens 2005). Populations inhabiting headwater stream ecosystems are especially sensitive to rainy conditions, as short and severe periods of drought could represent a high risk of extinction of their populations (Boulton 2003). The last decade in central and northern Mexico has been dryer than preceding decades (Seager et al. 2009): the most severe drought recorded from the BRSG was during 2010–2015, with the year 2012 being the most intense (Mendoza-Villa et al. 2018). Climatic predictions at a regional scale indicate that naturally occurring sub-decadal droughts will be made more frequent and widespread by anthropogenic climate change (Seager et al. 2009). Locally, water from the localities of the new species is intensively used for human consumption, crops, and livestock activities. Impacts driven by climate change are expected to be substantial on headwater streams ecosystems, which makes diagnosing and planning for conservation an urgent task (Durance and Ormerod 2007). From this perspective, the conservation of the headwaters of the rivers, as well as the maintenance of seasonal water regimes is of utmost importance to preserve endemic species, especially those that have very narrow distributions, such as *P. xihui*. Human actions also induce climate change to be faster in these areas, affecting the general ecological functioning of the Sierra and with it, also human activities (pers. obs.).

Collections for populations from the new species were made in the year 2002 and attempted in 2019, covering nearly 20 years. The climatic conditions and intense use of water described above has probably been related to the dramatic change observed by us at the visited sites, in which three of the five streams were almost dry or completely modified. In June 2019, an attempt to collect with the same sampling effort used in 2002 was carried out at all sites. We failed to find any crayfish at Las Camelinas, Saldiveña, and San Juanito, and in the remainder, crayfish were at much lower abundances than previously recorded. Additionally, several mass mortalities of crayfish were recorded from some sites, produced by the use of pesticides in crops surrounding the small streams.

As seen by their location, most populations were found in separated streams which were not in contact with each other for most of the year or even for several years. Most of individuals were found in such small populations and face situations of high dryness, in which they are limited to a small number of pools, representing a high risk of local extinctions. If crayfish diversity is one of the most endangered among freshwater fauna in the world (Richman et al. 2015), cambarids have the most threatened species in Mexico concerning freshwater Crustacea (Alvarez and Villalobos 2016). The new species is an especially sensitive case derived from its peculiar habitat and narrow distribution ranges, which emphasizes an urgent need to design and fulfill conservation measures in the short term to avoid extinction of most of its populations. Consequently, efforts to include the species into the Mexican law NOM-059-SEMARNAT-2010: Environmental Protection-Native species of Mexico of wild flora and fauna will be conducted.

## Supplementary Material

XML Treatment for Procambarus xihui

## Supplementary materials

Supplementary material 1 Results of the ABGD, bPTP and GMYC species delimitation analysis Data type Delimitation analyses https://binary.pensoft.net/file/563399This dataset is made available under the Open Database License (http://opendatacommons.org/licenses/odbl/1.0/). The Open Database License (ODbL) is a license agreement intended to allow users to freely share, modify, and use this Dataset while maintaining this same freedom for others, provided that the original source and author(s) are credited.Carlos Pedraza-Lara, Pedro Joaquín Gutiérrez-Yurrita, Vladimir Salvador De Jesus-Bonilla

Supplementary material 2 Table S1 Data type Genetic distances Explanation note Uncorrected *P*-distances for the COI fragment between species included in this study (in bold) and standard error. https://binary.pensoft.net/file/563400This dataset is made available under the Open Database License (http://opendatacommons.org/licenses/odbl/1.0/). The Open Database License (ODbL) is a license agreement intended to allow users to freely share, modify, and use this Dataset while maintaining this same freedom for others, provided that the original source and author(s) are credited.Carlos Pedraza-Lara, Pedro Joaquín Gutiérrez-Yurrita, Vladimir Salvador De Jesus-Bonilla
