# Supplementary material for: A new species of Procambarus (Decapoda, Cambaridae) from the State of Querétaro, Mexico
Source: Zookeys. 2021 Jul 6;1048:1–21. doi: 10.3897/zookeys.1048.57493 (PMC8277657; doi:10.3897/zookeys.1048.57493)
Supplement: Supplementary material 1 — Results of the ABGD, bPTP and GMYC species delimitation analysis [file zookeys-1048-001-s001.docx]

**Supplementary material 1**: Results of the ABGD, bPTP and GMYC species delimitation analysis.

#Result of ABGD species delimitation


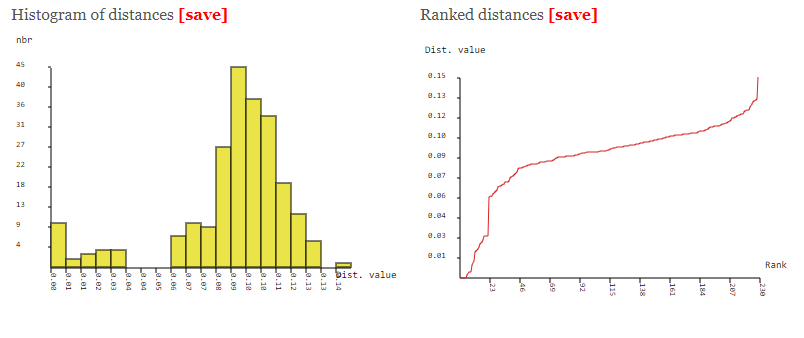


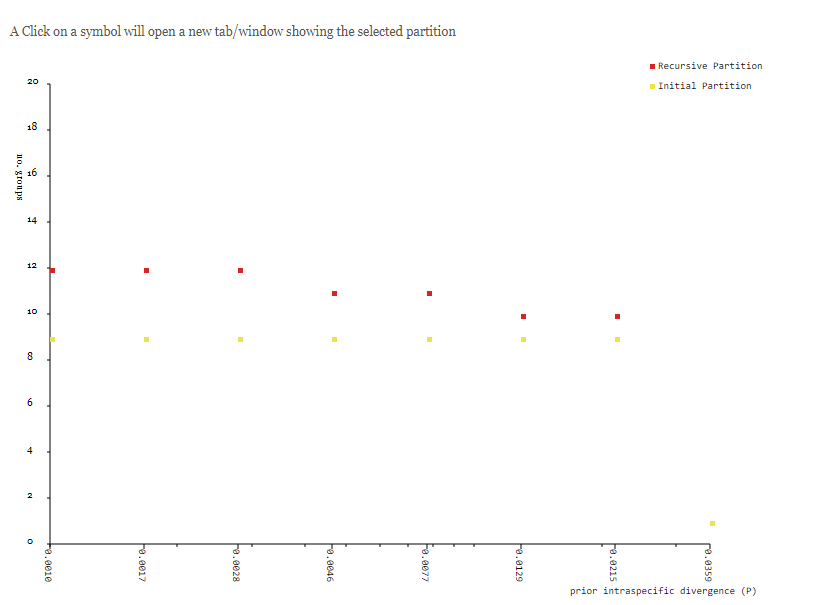


**Partition with prior maximal distance P=1.00e-03/P=1.67e-03/P=2.78e-03**
Distance JC69 Jukes-Cantor MinSlope=0.500000

**Group[ 1 ] n: 3 ;**id: Procambarus_toltecae_isolate_PopHui Procambarus_toltecae_CPLC3 Procambarus_toltecae_CPLC28
**Group[ 2 ] n: 2 ;**id: Procambarus_regiomontanus_DJ43 Procambarus_regiomontanus_CPLC4
**Group[ 3 ] n: 2 ;**id: Procambarus_acutus_3952Canalmante Procambarus_acutus_isolate_PopMan
**Group[ 4 ] n: 2 ;**id: Procambarus_caballeroi_CPL2420 Procambarus_caballeroi_CPL2419
**Group[ 5 ] n: 2 ;**id: Procambarus_villalobosi_CPLC11 Procambarus_villalobosi_CPLC33
**Group[ 6 ] n: 1 ;**id: Procambarus_gonopodocristatus_CPLC30
**Group[ 7 ] n: 1 ;**id: Procambarus_sp_CPLC27
**Group[ 8 ] n: 1 ;**id: Procambarus_digueti_CPLC12
**Group[ 9 ] n: 2 ;**id: Procambarus_strenti_CPLC10 Procambarus_roberti_CPLC32
**Group[ 10 ] n: 2 ;**id: Procambarus_cuevachicae_CPL2425 Procambarus_cuevachicae_CPL2424
**Group[ 11 ] n: 1 ;**id: Procambarus_hidalgoensis_CPLC5
**Group[ 12 ] n: 3 ;**id: Procambarus_sp_CPLC1 Procambarus_sp_CPLC23B Procambarus_sp_CPLC26B

**Partition with prior maximal distance P=4.64e-03/P=7.74e-03**
Distance JC69 Jukes-Cantor MinSlope=0.500000
Download (left click and save) or see below the tree file corresponding to this partition: click [**here**](https://bioinfo.mnhn.fr/abi/public/abgd/temp/9601.842242013/groupe4.tree)

**Group[ 1 ] n: 3 ;**id: Procambarus_toltecae_isolate_PopHui Procambarus_toltecae_CPLC3 Procambarus_toltecae_CPLC28
**Group[ 2 ] n: 2 ;**id: Procambarus_regiomontanus_DJ43 Procambarus_regiomontanus_CPLC4
**Group[ 3 ] n: 2 ;**id: Procambarus_acutus_3952Canalmante Procambarus_acutus_isolate_PopMan
**Group[ 4 ] n: 2 ;**id: Procambarus_caballeroi_CPL2420 Procambarus_caballeroi_CPL2419
**Group[ 5 ] n: 2 ;**id: Procambarus_villalobosi_CPLC11 Procambarus_villalobosi_CPLC33
**Group[ 6 ] n: 1 ;**id: Procambarus_gonopodocristatus_CPLC30
**Group[ 7 ] n: 4 ;**id: Procambarus_sp_CPLC27 Procambarus_sp_CPLC1 Procambarus_sp_CPLC23B Procambarus_sp_CPLC26B
**Group[ 8 ] n: 1 ;**id: Procambarus_digueti_CPLC12
**Group[ 9 ] n: 2 ;**id: Procambarus_strenti_CPLC10 Procambarus_roberti_CPLC32
**Group[ 10 ] n: 2 ;**id: Procambarus_cuevachicae_CPL2425 Procambarus_cuevachicae_CPL2424
**Group[ 11 ] n: 1 ;**id: Procambarus_hidalgoensis_CPLC5

**Partition with prior maximal distance P=1.29e-02/P=2.15e-02**
Distance JC69 Jukes-Cantor MinSlope=0.500000
Download (left click and save) or see below the tree file corresponding to this partition: click [**here**](https://bioinfo.mnhn.fr/abi/public/abgd/temp/9601.842242013/groupe6.tree)

**Group[ 1 ] n: 3 ;**id: Procambarus_toltecae_isolate_PopHui Procambarus_toltecae_CPLC3 Procambarus_toltecae_CPLC28
**Group[ 2 ] n: 2 ;**id: Procambarus_regiomontanus_DJ43 Procambarus_regiomontanus_CPLC4
**Group[ 3 ] n: 4 ;**id: Procambarus_acutus_3952Canalmante Procambarus_acutus_isolate_PopMan Procambarus_cuevachicae_CPL2425 Procambarus_cuevachicae_CPL2424
**Group[ 4 ] n: 2 ;**id: Procambarus_caballeroi_CPL2420 Procambarus_caballeroi_CPL2419
**Group[ 5 ] n: 2 ;**id: Procambarus_villalobosi_CPLC11 Procambarus_villalobosi_CPLC33
**Group[ 6 ] n: 1 ;**id: Procambarus_gonopodocristatus_CPLC30
**Group[ 7 ] n: 4 ;**id: Procambarus_sp_CPLC27 Procambarus_sp_CPLC1 Procambarus_sp_CPLC23B Procambarus_sp_CPLC26B
**Group[ 8 ] n: 1 ;**id: Procambarus_digueti_CPLC12
**Group[ 9 ] n: 2 ;**id: Procambarus_strenti_CPLC10 Procambarus_roberti_CPLC32
**Group[ 10 ] n: 1 ;**id: Procambarus_hidalgoensis_CPLC5

#Result of bPTP species delimitation


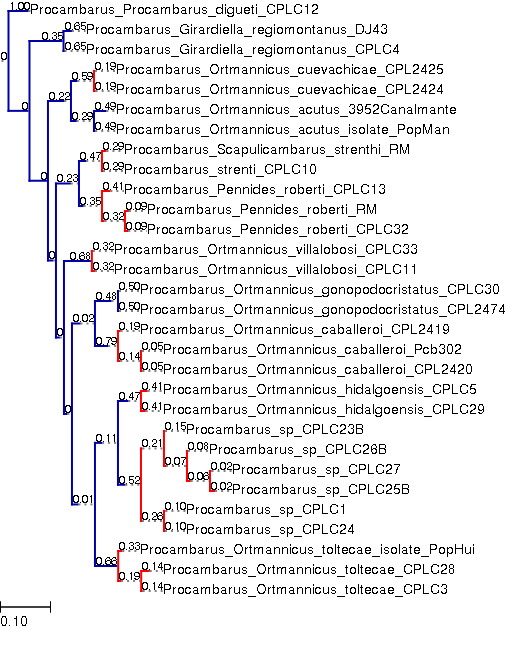


Most supported partition found by simple heuristic search

Species 1 (support = 1.000)

Procambarus_Procambarus_digueti_CPLC12

Species 2 (support = 0.680)

Procambarus_Ortmannicus_villalobosi_CPLC33,Procambarus_Ortmannicus_villalobosi_CPLC11

Species 3 (support = 0.664)

Procambarus_Ortmannicus_toltecae_CPLC3,Procambarus_Ortmannicus_toltecae_isolate_PopHui,Procambarus_Ortmannicus_toltecae_CPLC28

Species 4 (support = 0.790)

Procambarus_Ortmannicus_caballeroi_CPL2419,Procambarus_Ortmannicus_caballeroi_Pcb302,Procambarus_Ortmannicus_caballeroi_CPL2420

Species 5 (support = 0.474)

Procambarus_Scapulicambarus_strenthi_RM,Procambarus_strenti_CPLC10

Species 6 (support = 0.353)

Procambarus_Pennides_roberti_CPLC13,Procambarus_Pennides_roberti_RM,Procambarus_Pennides_roberti_CPLC32

Species 7 (support = 0.473)

Procambarus_Ortmannicus_hidalgoensis_CPLC5,Procambarus_Ortmannicus_hidalgoensis_CPLC29

Species 8 (support = 0.522)

Procambarus_sp_CPLC23B,Procambarus_sp_CPLC26B,Procambarus_sp_CPLC27,Procambarus_sp_CPLC25B,Procambarus_sp_CPLC1,Procambarus_sp_CPLC24

Species 9 (support = 0.586)

Procambarus_Ortmannicus_cuevachicae_CPL2425,Procambarus_Ortmannicus_cuevachicae_CPL2424

Species 10 (support = 0.501)

Procambarus_Ortmannicus_gonopodocristatus_CPLC30

Species 11 (support = 0.501)

Procambarus_Ortmannicus_gonopodocristatus_CPL2474

Species 12 (support = 0.491)

Procambarus_Ortmannicus_acutus_3952Canalmante

Species 13 (support = 0.491)

Procambarus_Ortmannicus_acutus_isolate_PopMan

Species 14 (support = 0.654)

Procambarus_Girardiella_regiomontanus_DJ43

Species 15 (support = 0.654)

Procambarus_Girardiella_regiomontanus_CPLC4

#Result of GMYC species delimitation


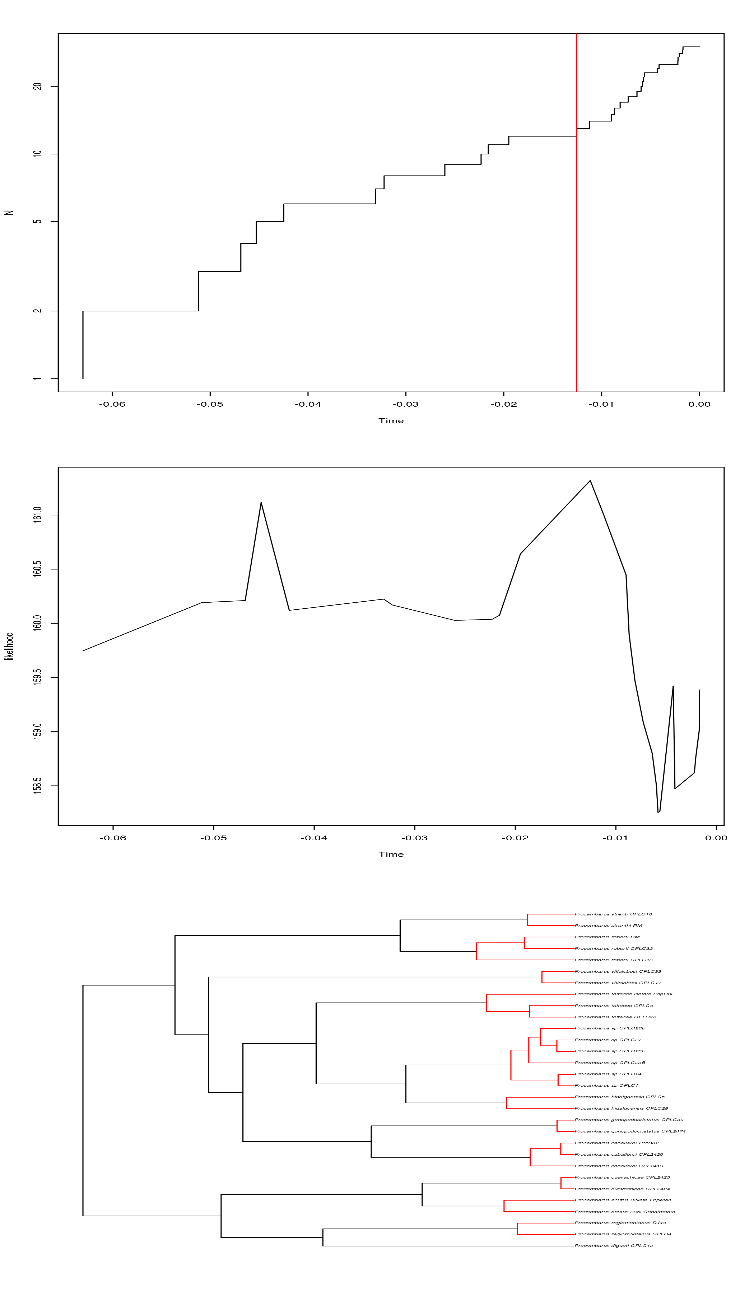


method: single
likelihood of null model: 159.7461
maximum likelihood of GMYC model: 161.3187
likelihood ratio: 3.145313
result of LR test: 0.2074933n.s.

number of ML clusters: 11
confidence interval: 1-11

number of ML entities: 12
confidence interval: 1-29

threshold time: -0.01254787
