# Supplementary material for: A new species of Procambarus (Decapoda, Cambaridae) from the State of Querétaro, Mexico
Source: Zookeys. 2021 Jul 6;1048:1–21. doi: 10.3897/zookeys.1048.57493 (PMC8277657; doi:10.3897/zookeys.1048.57493)
Supplement: Supplementary material 2 — Table S1 [file zookeys-1048-001-s002.docx]

**Supplementary material 2. Genetic distances. *P*-uncorrected distances for the COI fragment between species included in this study (in bold) and standard error.**

|  |  | 1 |  | 2 |  | 3 |  | 4 |  | 5 |  | 6 |  | 7 |  | 8 |  | 9 |  | 10 |  | 11 |  |
| --- | --- | --- | --- | --- | --- | --- | --- | --- | --- | --- | --- | --- | --- | --- | --- | --- | --- | --- | --- | --- | --- | --- | --- |
| 1 | *Procambarus xihui* sp. nov. |  |  |  |  |  |  |  |  |  |  |  |  |  |  |  |  |  |  |  |  |  |  |
| 2 | *Procambarus hidalgoensis* | **0.030** | 0.011 |  |  |  |  |  |  |  |  |  |  |  |  |  |  |  |  |  |  |  |  |
| 3 | *Procambarus toltecae* | **0.082** | 0.012 | **0.087** | 0.017 |  |  |  |  |  |  |  |  |  |  |  |  |  |  |  |  |  |  |
| 4 | *Procambarus caballeroi* | **0.082** | 0.012 | **0.091** | 0.018 | **0.088** | 0.013 |  |  |  |  |  |  |  |  |  |  |  |  |  |  |  |  |
| 5 | *Procambarus gonopodocristatus* | **0.088** | 0.013 | **0.094** | 0.017 | **0.102** | 0.014 | **0.066** | 0.011 |  |  |  |  |  |  |  |  |  |  |  |  |  |  |
| 6 | *Procambarus villalobosi* | **0.088** | 0.012 | **0.098** | 0.018 | **0.086** | 0.012 | **0.094** | 0.012 | **0.086** | 0.013 |  |  |  |  |  |  |  |  |  |  |  |  |
| 7 | *Procambarus roberti* | **0.064** | 0.010 | **0.059** | 0.015 | **0.086** | 0.012 | **0.086** | 0.011 | **0.077** | 0.012 | **0.078** | 0.012 |  |  |  |  |  |  |  |  |  |  |
| 8 | *Procambarus strenthi* | **0.059** | 0.010 | **0.073** | 0.016 | **0.075** | 0.012 | **0.089** | 0.012 | **0.078** | 0.012 | **0.080** | 0.012 | **0.028** | 0.007 |  |  |  |  |  |  |  |  |
| 9 | *Procambarus acutus* | **0.083** | 0.012 | **0.068** | 0.015 | **0.090** | 0.011 | **0.096** | 0.012 | **0.093** | 0.013 | **0.095** | 0.012 | **0.083** | 0.011 | **0.080** | 0.012 |  |  |  |  |  |  |
| 10 | *Procambarus cuevachicae* | **0.090** | 0.012 | **0.070** | 0.016 | **0.099** | 0.013 | **0.093** | 0.012 | **0.093** | 0.014 | **0.097** | 0.013 | **0.087** | 0.011 | **0.090** | 0.012 | **0.023** | 0.006 |  |  |  |  |
| 11 | *Procambarus regiomontanus* | **0.106** | 0.013 | **0.109** | 0.018 | **0.119** | 0.014 | **0.097** | 0.013 | **0.098** | 0.013 | **0.104** | 0.013 | **0.099** | 0.011 | **0.100** | 0.012 | **0.108** | 0.012 | **0.107** | 0.012 |  |  |
| 12 | *Procambarus digueti* | **0.101** | 0.013 | **0.098** | 0.019 | **0.107** | 0.014 | **0.114** | 0.013 | **0.114** | 0.015 | **0.108** | 0.013 | **0.112** | 0.013 | **0.101** | 0.013 | **0.102** | 0.012 | **0.106** | 0.012 | **0.095** | 0.012 |
